# Cutaneous Nocardiosis in the Very Elderly: A Case of Lymphocutaneous Infection and a Literature Review of Patients Aged Over 85 Years

**DOI:** 10.7759/cureus.87933

**Published:** 2025-07-14

**Authors:** Natsumi Shimada, Natsuko Saito-Sasaki, Etsuko Okada, Yu Sawada

**Affiliations:** 1 Dermatology, University of Occupational and Environmental Health, Kitakyushu, JPN

**Keywords:** case report, literature review, literature review of disease, nocardia, nocardia species

## Abstract

Cutaneous nocardiosis is an uncommon but clinically significant opportunistic infection, primarily affecting immunocompromised individuals, including those with underlying malignancies, organ transplants, or chronic corticosteroid use. Although it can also occur in elderly patients, this is often due to age-associated immunosenescence or comorbid conditions that impair immune function. We describe a case of lymphocutaneous *Nocardia brasiliensis* infection in an 87-year-old man with no history of trauma or corticosteroid use. The patient had been receiving long-term low-dose methotrexate (MTX) for rheumatoid arthritis. He presented with painful, erythematous nodules arranged along the lymphatic vessels of the right forearm. Histopathological analysis and culture confirmed the diagnosis. Oral trimethoprim-sulfamethoxazole (TMP-SMX) and minocycline were initiated; minocycline was discontinued after three weeks due to gastrointestinal symptoms. MTX was also stopped in response to clinical progression. The lesions resolved completely with continued TMP-SMX monotherapy over a three-month course. A brief review of the literature revealed only a few reported cases of cutaneous nocardiosis in individuals over 85 years of age. Some of these lacked clear immunosuppressive backgrounds or trauma history. While TMP-SMX remains the standard therapy, its use in older adults may be constrained by tolerability. This case serves as a reminder that *Nocardia* infection should be considered in the differential diagnosis of nodular skin lesions in very elderly patients, even in the absence of typical risk factors.

## Introduction

*Nocardia* species are aerobic actinomycetes that can cause opportunistic infections, including pulmonary, central nervous system, and cutaneous disease [1]. Cutaneous nocardiosis is relatively rare and typically affects immunocompromised individuals or those with trauma exposure [2,3]. However, aging is increasingly recognized as a risk factor for infection due to immune system suppression [4,5]. We report a case of cutaneous lymphangitic nocardiosis caused by *Nocardia brasiliensis* in an 87-year-old man with no history of immunosuppressive therapy, and we also provide a literature review of cutaneous *Nocardia* infections in patients aged over 85.

## Case presentation

**Figure 1:**
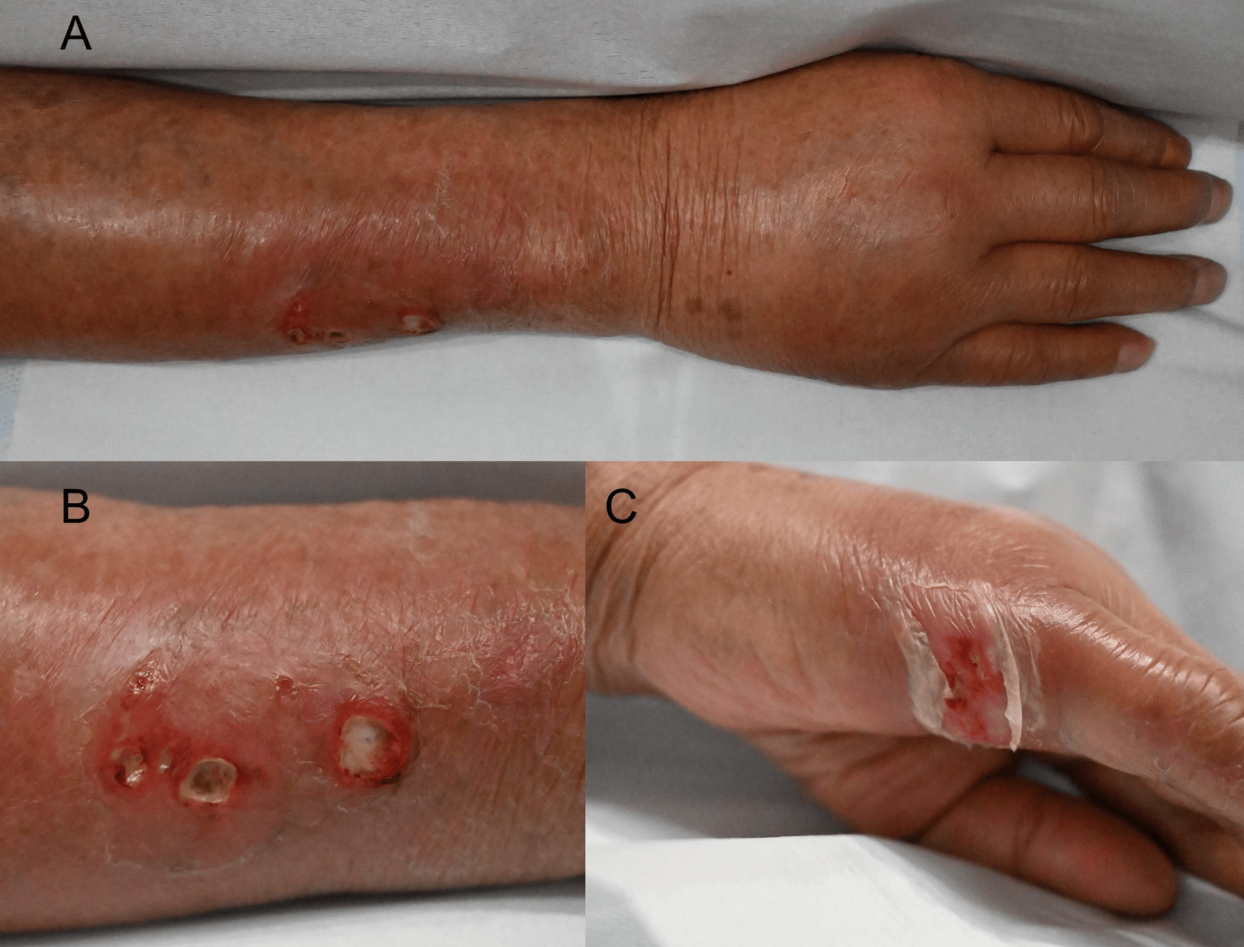
Clinical presentation of the right upper extremity at the initial visit (A) Multiple erythematous nodules arranged in a linear pattern along the lymphatic vessels of the right forearm. (B) Close-up view showing dome-shaped, ulcerated nodules with purulent discharge. (C) Erythematous erosion on the dorsal hand, initially appearing as the first lesion.

An 87-year-old Japanese male presented to our Dermatology Department with a 10-day history of painful erythematous nodules on his right forearm. The lesions initially appeared as a single red nodule on the dorsum of the right hand and gradually spread proximally along the lymphatic channels, forming a linear array. Over time, the nodules spontaneously ruptured and developed into ulcers. He denied any history of trauma, insect bites, animal contact, or soil exposure. His medical history included rheumatoid arthritis, for which he had been taking methotrexate (MTX) continuously for seven years, as well as well-controlled type 2 diabetes mellitus and hypertension. He had no history of malignancy, collagen vascular diseases, or the use of corticosteroids or biologics. On physical examination, multiple erythematous, firm, dome-shaped nodules were observed along the lymphatic drainage pathway of the right forearm, consistent with a lymphangitic distribution (Figure 1). The lesions were mildly tender to palpation. No fever, weight loss, or lymphadenopathy was noted. Laboratory tests, including complete blood count, liver enzymes, and renal function, were within normal limits. In cases of cutaneous lesions with a sporotrichoid distribution, differential diagnoses include sporotrichosis, atypical mycobacterial infection, nocardiosis, and cutaneous leishmaniasis. Culture of a skin swab from the ulcerated area identified *N. brasiliensis*.

**Figure 2:**
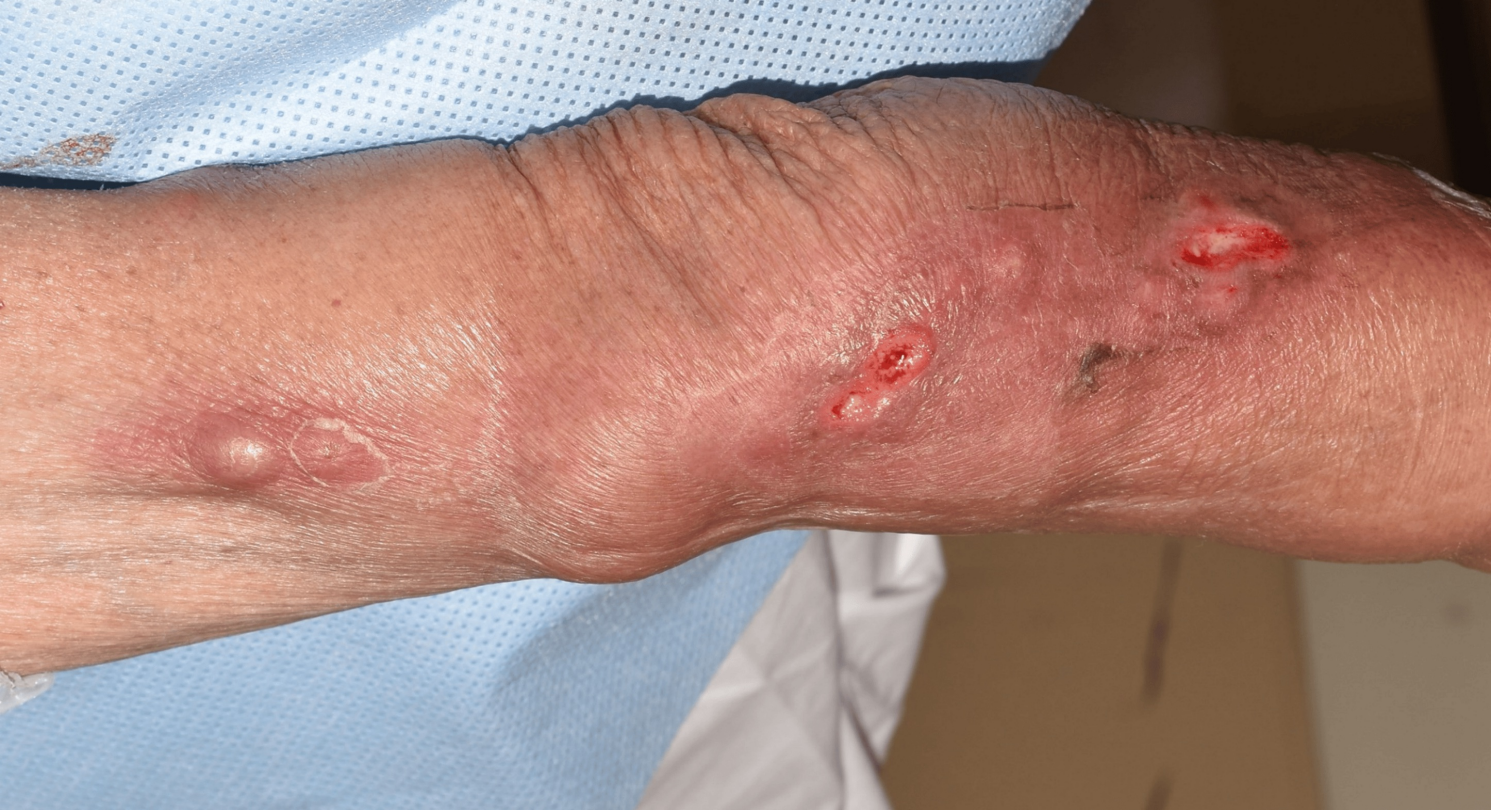
Clinical course on day 11 after starting antimicrobial therapy Despite combination therapy with TMP-SMX and minocycline, the lesions extended proximally with new subcutaneous abscess formation on the upper arm, indicating disease progression. TMP-SMX: trimethoprim-sulfamethoxazole

Following the diagnosis, the patient was started on oral trimethoprim-sulfamethoxazole (TMP-SMX; 160 mg/800 mg twice daily) and minocycline (100 mg once daily). However, despite antibiotic therapy, the nodular lesions continued to spread proximally along the lymphatic channels, and by day 11, a new subcutaneous abscess had developed on the upper arm (Figure 2). Given the clinical progression, MTX was discontinued on day 21 to reduce immunosuppression and promote infection control. Around the same time, the patient developed gastrointestinal symptoms, including diarrhea, attributed to minocycline. As a result, minocycline was also discontinued, and TMP-SMX was continued as monotherapy.

**Figure 3:**
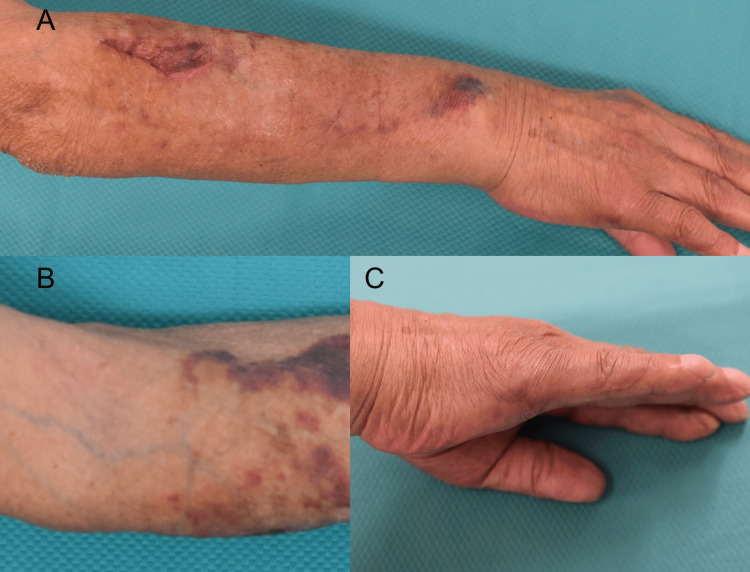
Clinical outcome after three months of treatment with TMP-SMX monotherapy (A) Healed ulcer with post-inflammatory scarring on the forearm. (B) Residual pigmentation and post-inflammatory changes along the lymphatic route. (C) Complete resolution of the initial lesion on the dorsum of the hand without scarring. TMP-SMX: trimethoprim-sulfamethoxazole

The lesions gradually improved under TMP-SMX alone. By the end of a three-month treatment course, the nodules had completely resolved (Figure 3).

## Discussion

Cutaneous nocardiosis is an uncommon but clinically relevant infection, particularly among the very elderly [6]. Although previous studies have reported that patients over the age of 55 are more likely to develop severe disease, it remains unclear whether this also applies to the very elderly population [7]. While nocardiosis is traditionally associated with immunocompromised states such as malignancy, organ transplantation, and long-term corticosteroid use, the present case illustrates that advanced age, particularly over 85 years, in combination with an underlying immunosuppressive condition such as malignancy, may predispose individuals to opportunistic infections such as *N. brasiliensis *[7,8].

Our patient was receiving MTX for rheumatoid arthritis at the time of presentation. Although MTX had been well tolerated for years, the skin lesions showed progressive lymphatic dissemination despite early antibiotic therapy. This clinical course prompted discontinuation of MTX on day 21 to reduce immunosuppressive pressure and support infection control. This case exemplifies the importance of re-evaluating immunosuppressive regimens in the context of emerging opportunistic infections, especially in the oldest-old population.

We also reviewed five previously reported cases of cutaneous nocardiosis in patients over 85 years of age [9-13]. Among them, three had no documented use of immunosuppressive therapy but had either ongoing or recent exposure. These findings highlight that immunosenescence alone, characterized by age-related declines in T-cell function, neutrophil chemotaxis, and monocyte regulation, may permit the development of nocardial infection. When coupled with even modest immunosuppressive therapy, the risk likely increases synergistically.

In previously reported cases, trauma was often implicated as a portal of entry for *Nocardia* species [2,3]. However, in our case, no clear history of skin injury, insect bite, or soil exposure was identified, underscoring that even in the absence of an identifiable entry point, subclinical barrier disruptions in the elderly may suffice to initiate infection.

TMP-SMX remains the mainstay of treatment for cutaneous nocardiosis and was used in four of the five reviewed cases [9-13]. However, its use in older adults must be approached with caution due to risks of renal toxicity, hyperkalemia, and bone marrow suppression. In one prior case, leukopenia necessitated discontinuation of TMP-SMX [9]. In our case, initial combination therapy with TMP-SMX and minocycline was employed; however, minocycline had to be stopped due to gastrointestinal side effects. The patient subsequently achieved full clinical resolution with TMP-SMX monotherapy over a three-month period.

Taken together, these findings highlight the need to consider cutaneous nocardiosis in patients over 85 years old, especially those with underlying conditions or on immunomodulatory therapy. Early diagnosis using stains and culture is essential. Treatment should balance effectiveness, tolerability, and kidney function in this age group.

## Conclusions

Cutaneous lymphangitic nocardiosis should be considered in elderly patients presenting with nodular skin lesions along lymphatic channels, even in the absence of trauma or overt immunosuppression. Advanced age, combined with mild immunosuppressive therapy such as MTX, may increase the risk of infection. Early diagnosis, appropriate culture-based identification, and individualized antibiotic management, such as balancing efficacy and tolerability, are essential for favorable outcomes in this age population.
